# A Smartphone App to Facilitate Remote Patient-Provider Communication in Hearing Health Care: Usability and Effect on Hearing Aid Outcomes

**DOI:** 10.1089/tmj.2019.0109

**Published:** 2020-06-03

**Authors:** Elizabeth Convery, Gitte Keidser, Margot McLelland, Jennifer Groth

**Affiliations:** ^1^ National Acoustic Laboratories, Sydney, Australia.; ^2^ GN Hearing, Glenview, Illinois, USA.

**Keywords:** e-health, m-health, telehealth, rehabilitation

## Abstract

**Background:** Patients often need multiple fine-tuning appointments with their hearing health care provider to achieve satisfactory hearing aid outcomes. A smartphone app that enables patients to remotely request and receive new hearing aid settings could improve hearing health care access and efficiency.

**Introduction:** We assessed the usability of ReSound Assist™, (ReSound America, Bloomington, MN) the remote communication feature of a hearing aid app, and investigated whether hearing aid outcomes are influenced by app-based versus in-person patient-provider communication.

**Materials and Methods:** Thirty adults were fit bilaterally with hearing aids and randomized to intervention and control groups. During a 6-week field trial, participants reported hearing aid problems via ReSound Assist (intervention) or at a scheduled face-to-face follow-up appointment (control). Usability of ReSound Assist was assessed with a questionnaire and interview. Hearing aid performance, benefit, satisfaction, and daily usage were compared for both groups.

**Results:** ReSound Assist was rated as highly usable. Participants identified specific aspects of effectiveness and efficiency that could be improved. Similar problems were reported by intervention and control participants regardless of communication mode (app-based vs. in-person). However, almost half the requests received via ReSound Assist were for problems that required advice from the provider or physical modifications to the hearing aids rather than fine-tuning, highlighting the continued importance of in-person hearing health care. There was no significant difference in hearing aid outcomes between intervention and control participants.

**Conclusions:** Apps enabling remote patient-provider communication are a viable method for hearing aid users to seek and receive help with hearing aid problems that can be addressed through fine-tuning.

## Introduction

Hearing loss, a disorder of the ear characterized by a reduction in auditory sensitivity, is the most prevalent sensory impairment^1^ and the third leading contributor to years lived with disability worldwide.^2,3^ Hearing aids are the most common form of rehabilitation provided to adults with hearing loss and are a cost-effective intervention^4,5^ that reduce activity limitations and participation restrictions and improve health-related quality of life.^6,7^ Hearing aids are fit to individual patients' needs by applying a prescriptive formula to their hearing thresholds.^8^ While the most widely used formulas have been empirically validated, they yield hearing aid settings that address the needs of the average patient, which are not necessarily preferred by the individual.^9^ As a result, hearing aids often need to be fine-tuned by the provider to ensure optimal and satisfactory speech understanding, sound quality, and comfort in a range of acoustic environments. Fine-tuning may be undertaken at the time of the initial hearing aid fitting, or, more commonly, after the patient has had the opportunity to wear the hearing aids in daily life. For this reason, several follow-up appointments may be needed to meet a patient's individual preferences.

The potential need for multiple face-to-face follow-up appointments poses a number of challenges to hearing health care provision. First, patients who have mobility problems, are time-poor, or do not live near an audiology clinic can find it difficult to make repeated in-person visits to a provider.^10^ As a result, they may delay or forgo seeking help for their hearing aid problems. Second, patients can struggle to accurately describe listening problems retrospectively.^11^ If there is a lengthy delay between their experience of a problem and a visit to their provider, key details about the problem may be forgotten. Third, hearing aids are typically fine-tuned in the clinic, with the patient expected to rapidly assess whether the new, fine-tuned settings have adequately addressed the problem. This may lead to an unsatisfactory result when the patient trials the new settings in daily life, particularly if the original problem was experienced in an acoustic environment that differs markedly from a quiet clinic.^12^ Fourth, a recent longitudinal study of a large hearing health care provider found that unplanned fine-tuning appointments made up the largest proportion of appointment types, with almost a third of their patients attending four or more fine-tuning appointments after a hearing aid fitting.^13^ Together, these challenges suggest that postfitting care is a logical target for improving hearing health care access, effectiveness, and efficiency. Hearing aid manufacturers have recently begun to leverage cloud-based m-health technologies, such as smartphone applications (apps) that enable remote communication between patients and providers, in an effort to achieve these goals.^14,15^

Before m-health innovations are implemented into routine clinical practice, they must be rigorously evaluated to ensure they are both usable by, and beneficial for, the target patient population. The concept of usability encompasses three major components: (1) effectiveness, the accuracy and completeness with which the technology can be used to accomplish its goal; (2) efficiency, the resources expended by the user relative to the technology's effectiveness; and (3) satisfaction, the degree to which users are comfortable with, and accepting of, the technology.^16^ A recent study found that the usability of several m-health apps for chronic condition self-management was suboptimal across each of these dimensions, with excessive navigation through multiple screens, complex language, and ambiguous instructions identified as barriers to use.^17^ While m-health technologies are intended to improve health care access and efficiency, the authors point out that poor app usability can actually introduce an additional obstacle to achieving this goal. Balancing health care efficiency with patient outcomes is another important consideration in the development of m-health technologies. If an existing element of service delivery is to be augmented or replaced with an app, it is critical to ensure that patient outcomes are at least equivalent to those achieved through standard face-to-face care.

An app enabling remote communication between patients and providers was recently introduced by hearing aid manufacturer GN Hearing. Since the app is the first of its kind, very little is known about its acceptability to patients and its effect on hearing rehabilitation outcomes. An exploratory study was therefore conducted to gather preliminary information about the feasibility of incorporating the app into clinical practice. The aims of the study were to (1) assess the usability of the remote communication feature of the app; and (2) determine whether hearing aid fitting outcomes are influenced by the mode of patient-provider communication.

## Materials and Methods

### Participants

Thirty adults (16 male, 14 female) took part in the study. The inclusion criteria were as follows: (1) ≤85 years of age; (2) a four-frequency average hearing loss (average of pure-tone thresholds at 0.5, 1, 2, and 4 kHz across both ears) between 25 and 75 dB HL; (3) smartphone ownership, to ensure data were collected on a sample that parallels likely real-world users of a hearing aid app; and (4) ≥1 year of bilateral hearing aid experience. The exclusion criteria were as follows: (1) presence of active ear disease; (2) non-English speaking; and (3) additional disabilities, such as severe cognitive impairment, that would preclude participation in the study. The median age of the participants was 67 years (range = 22–83 years). The median four-frequency average hearing loss was 45 dB HL (range = 29–75 dB HL).

### Hearing Aids and Smartphone App

The hearing aids used in the study were ReSound LiNX 3D™ 962 hearing aids (ReSound America, Bloomington, MN), receiver-in-ear devices with 4 programs, 17 channels, an environmental classifier, and binaural adaptive noise management algorithms. The smartphone app was the ReSound Smart 3D hearing aid app, which communicates with the user's hearing aids via a direct Bluetooth connection between the hearing aids and the user's smartphone. The app feature under test, ReSound Assist, enables hearing aid users to remotely request adjustments to their settings and to receive and upload the new settings from their provider. To use ReSound Assist, users must first have an internet connection. The user is then prompted to answer a series of questions to identify the nature of the problem, the environment(s) in which the problem is occurring, and the perceived severity of the problem. The request is sent to the provider via the cloud and the user receives an automated message indicating the approximate timeframe within which a response can be expected. In response to the request, the provider makes changes to the hearing aid settings within the fitting software and sends the new settings to the user via the cloud. The user is prompted in the app to download the new settings and upload them to the hearing aids.

### Usability Outcome Measures

#### ReSound Assist usability

Usability of ReSound Assist was assessed only in the intervention group with a modified version of the Telehealth Usability Questionnaire.^18^ In its original form, the questionnaire contains 21 items that assess acceptance of and ability to use telehealth services and equipment. In the modified version used in this study, the phrase *the telehealth system* was replaced with *the Assist feature in the ReSound Smart 3D app*. Three items probing ease of real-time communication with the provider (talking to, hearing, and seeing the provider via videoconferencing) were not relevant to ReSound Assist and were therefore removed. Possible scores range from 1 to 10, with lower ratings indicating greater usability.

#### Exit interview

Usability was further assessed in the intervention group during a semi-structured exit interview. The first six questions, which required participants to provide a rating on a 5-point Likert scale, probed ease of use, satisfaction with the questions and answer choices provided by the app, satisfaction with the new settings sent by the provider, preference for ReSound Assist versus the type of postfitting face-to-face consultation they have attended with their own hearing health care provider, and preference for a similar feature with their own hearing aids. The other two questions were open-ended and asked participants to describe the problem(s) they reported via ReSound Assist and their overall experience with the feature.

### Hearing Aid Outcome Measures

#### Hearing aid benefit

Hearing aid benefit was assessed with the Abbreviated Profile of Hearing Aid Benefit, a 24-item self-report inventory in which participants rate the degree of difficulty they experience in a variety of quiet, noisy, and reverberant environments.^19^

#### Hearing aid satisfaction

Hearing aid satisfaction was assessed with the 15-item Satisfaction with Amplification in Daily Life scale.^20^ Participants were asked to rate their satisfaction with device performance, effect on self-image, and negative aspects of hearing aid management, with higher ratings representing greater perceived satisfaction.

#### Speech understanding in noise

The signal-to-noise ratio at which 50% speech recognition in noise is achieved was measured with the Beautifully Efficient Speech Test.^21^ Testing was conducted in a circular array of 16 loudspeakers. Speech (subject-verb-object sentences containing three to eight morphemes) was presented from the loudspeaker at 0° azimuth; a recording of café noise was presented from the other 15 loudspeakers. The level of the speech was varied adaptively according to the participant's response (increased after an incorrect response, decreased after a correct response) and the level of the noise was fixed. Speech was presented adaptively until a minimum of 16 sentences had been presented and a test-retest standard error of 0.8 dB was reached, or a maximum of 32 sentences had been administered.^22^

#### Hearing aid usage

Hours of use were logged by the hearing aids and read out in the hearing aid fitting software at the end of the study.

### Procedure

Participants were assigned to one of two groups (intervention or control) matched for gender, age, and hearing loss severity. All participants attended an initial assessment at the laboratory during which otoscopy and pure tone air- and bone-conduction audiometry were completed and demographic data were recorded. At the second appointment, ∼2 weeks later, all participants were fitted bilaterally with the hearing aids and given the ReSound Smart 3D app. During the following 6-week field trial, intervention participants had access to ReSound Assist. Control participants did not have access to ReSound Assist; instead, they attended a face-to-face follow-up appointment 2 weeks postfitting. At the end of the field trial, all participants completed the outcome measures; intervention participants additionally completed the measures of ReSound Assist usability. The timing of the study appointments and the use of a 6-week field trial parallel real-world clinical practice in audiology, in which patients attend an initial assessment, are fitted with hearing aids ∼2 weeks later, and are followed up ∼6 weeks postfitting.

The treatment of participants was approved by the Australian Hearing Human Research Ethics Committee (AHHREC2018-18) and conformed in all respects to the Australian government's National Statement on Ethical Conduct in Human Research.^23^

## Results

### Usability of ReSound Assist

Twelve of the 15 intervention participants used the ReSound Assist feature at least once during the field trial. Of the participants who used ReSound Assist, 11 were successful, meaning they were able to access the feature, answer the prompt questions, send a request, and upload the new settings to their hearing aids. The one unsuccessful participant attempted to use ReSound Assist several times but received a “service unavailable” message each time. The problem could not be reproduced in the laboratory. Since the usability measures could only be completed by participants who accessed ReSound Assist, there is no Telehealth Usability Questionnaire^17^ data available for this participant, nor could he answer the closed-ended interview questions.

The mean overall score among the 11 participants who accessed ReSound Assist was 1.9 (standard deviation = 0.83), suggesting that they believed ReSound Assist was simple to use, they could use it to explain their needs effectively, and that it was an acceptable way to receive hearing health care services.

**FIG. 1.**
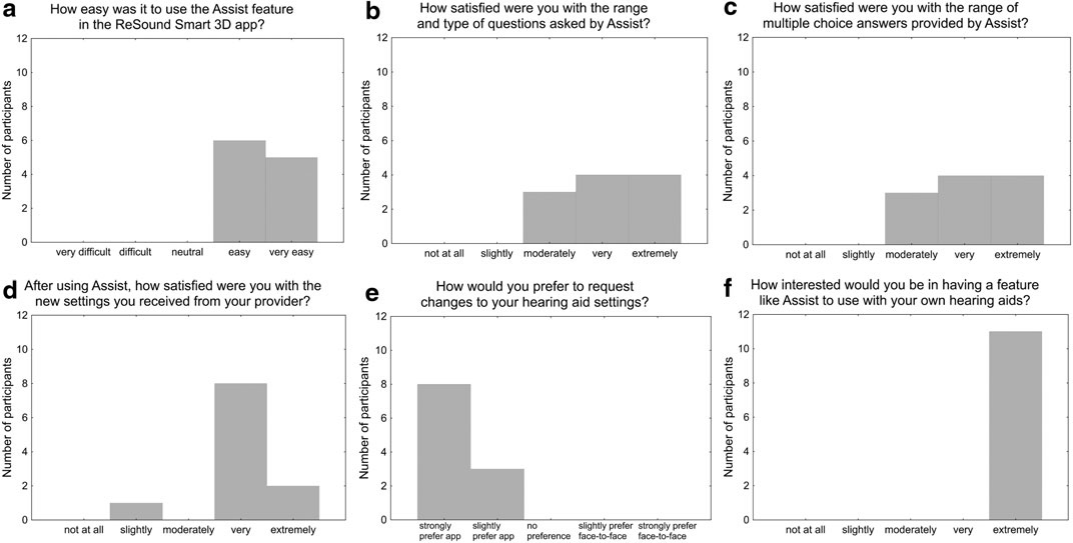
**(a-f)** Responses to the exit interview questions by the users of ReSound Assist™ (*n* = 11).

Responses to the closed-ended exit interview questions are shown in *Figure 1*. Overall, the 11 participants who accessed ReSound Assist rated the feature as highly usable, were satisfied with its question and answer options and the new settings they received from their provider, and reported a preference for app-based versus face-to-face postfitting patient-provider communication.

Responses to the open-ended questions about participants' experiences using ReSound Assist were classified according to the three components of usability: effectiveness, efficiency, and satisfaction. There were 14 comments about effectiveness (4 positive, 10 negative); 6 about efficiency (2 positive, 4 negative), and 6 about satisfaction (4 positive, 2 negative). Representative comments about each component are shown in *Table 1*. The preponderance of negative comments about the ReSound Assist's effectiveness and efficiency related primarily to the multiple-choice questions asked by the app to determine the nature and severity of the hearing aid user's problem. Several participants reported that their problem was not adequately covered by the answer choices, leading to concerns they were not clearly communicating the problem to the provider (effectiveness). The majority of participants felt the provider would only understand their problem if they added a written description in the text box in addition to answering the multiple-choice questions (efficiency). Participants were largely positive about the app's appearance and interface (satisfaction).

**Table 1.** Representative Comments Made by the Intervention Participants Who Used or Attempted to Use ReSound Assist™ (*n* = 12), Classified According to the Three Dimensions of Usability: Effectiveness, Efficiency, and Satisfaction

USABILITY DIMENSION	PARTICIPANT COMMENTS	
	POSITIVE	NEGATIVE
Effectiveness	“I managed to install the new settings without difficulty”	“When it worked it worked great, but it didn't work for me all the time due to connection issues”
	“It's handy to be able to add your own message at the end”	“I had to choose ‘other’ as my answer to many of the questions since my issue was not covered by the questions that were asked”
Efficiency	“I didn't use it initially as I expected it to be more convoluted, but it was surprisingly easy to do”	“It's easier to put my problem in an email rather than ticking boxes and hoping the predefined categories cover your problem”
	“It was simple to learn how to use it, even for me who is not that into technology”	“I saw an alert [about new settings] pop up on my screen, but it only flashed up for a short time, so I had to search for it”
Satisfaction	“Red on black is very easy to see”	“Red on black is hard to read”
	“I liked the look of the app. It didn't look like a game, so it wouldn't be overly enticing for others to look at, say, in a meeting”	“I'm used to Apple computers, so I found that using the ‘x’ to close the screen and go back took a little getting used to, as more of a PC feature”

The problems reported via ReSound Assist are shown in *Table 2*. Participants reported a total of 23 problems during the 6-week field trial. Twelve of the problems could be resolved via remote fine-tuning of the hearing aid settings; the other 11 required that advice be given, either via the app's message box, email, or telephone, or that the participant attend for a face-to-face consultation.

**Table 2.** Hearing Aid Problems Reported During the Field Trial by the Intervention Participants Who Successfully Used ReSound Assist (*n* = 11)

PROBLEMS THAT COULD BE ADDRESSED WITH HEARING AID FINE-TUNING	PROBLEMS THAT COULD NOT BE ADDRESSED WITH HEARING AID FINE-TUNING
Overall volume too soft or loud (6)	Cannot maintain Bluetooth connection between hearing aids and smartphone (5)
Acoustic feedback (2)	Cannot stream audio from smartphone to hearing aids (3)
Too much high-frequency emphasis (2)	Itchy ear canals (2)
Alert beeps too loud (1)	Uncomfortable physical fit of hearing aids in ear canal (1)
Would like a telecoil program (1)	

Number of participants reporting each problem shown in parentheses.

Three participants did not use ReSound Assist. All reported they did not experience any problems with their hearing aids that would warrant contacting their provider.

### Hearing Aid Fitting Outcomes

Outcome measure data were assessed to ensure they met the necessary assumptions for performing independent samples *t*-tests, namely a lack of influential outliers, normality of distribution, and homogeneity of variances.^24^ *Table 3* shows the mean, standard deviation, and range for each outcome measure and the results of the independent samples *t*-test comparing the intervention and control groups. One participant in the control group did not complete speech discrimination testing because he lost one of the hearing aids during the trial. Hours of use could not be downloaded from the hearing aids of one participant in the intervention group. There were no significant differences (all *p* > 0.05) between the intervention and control groups in terms of speech discrimination threshold, hearing aid benefit, hearing aid satisfaction, or hours of daily hearing aid usage.

**Table 3.** The Mean, Standard Deviation, and Range of Each Outcome Measure Variable for the Two Participant Groups

VARIABLE	INTERVENTION GROUP (N = 15)		CONTROL GROUP (N = 15)		t	p
	MEAN (SD)	RANGE	MEAN (SD)	RANGE		
Speech reception threshold (dB SNR)	−4.6 (3.5)	−8.3 to 3.2	−3.9 (2.8)	−7.6 to 2.1	−0.52	0.61
APHAB ease of communication	11.4 (7.3)	1–25	15.2 (7.9)	1–29	−1.33	0.19
APHAB background noise	35.2 (22.3)	7–89	29.6 (15.6)	5–62	0.79	0.43
APHAB reverberation	32.6 (19.8)	9–77	26.2 (15.6)	1–52	0.99	0.33
APHAB aversiveness	38.2 (23.3)	3–89	29.0 (22.9)	1–75	1.09	0.29
APHAB global score	26.3 (14.8)	7–62	23.6 (12.4)	2–47	0.55	0.59
SADL positive effect	5.5 (0.85)	3.5–6.7	5.8 (0.81)	4.2–6.8	−0.80	0.43
SADL negative features	5.4 (0.90)	3.7–7.0	5.3 (0.80)	3.7–6.3	0.32	0.75
SADL personal image	6.4 (0.44)	5.7–7.0	6.5 (0.43)	5.7–7.0	−0.42	0.68
SADL global score	5.7 (0.60)	4.1–6.6	5.9 (0.56)	4.7–6.6	−0.57	0.58
Average daily use (h)	10.7 (6.1)	3–24	8.9 (5.4)	2–15	0.84	0.41

APHAB, Abbreviated Profile of Hearing Aid Benefit; dB SNR, decibel signal-to-noise ratio; SADL, satisfaction with amplification in daily life; SD, standard deviation.

## Discussion

m-Health apps that enable remote patient-provider communication are a potential way to increase the accessibility of hearing health care and to facilitate real-time reporting of hearing aid problems. Usability has been identified in previous studies as an important prerequisite to successful integration of m-health apps into routine clinical practice.^17,25^ In the present study, the majority of ReSound Assist users successfully used the feature at least once during the trial and rated their satisfaction with ReSound Assist and its usability very positively.

In line with Sarkar et al.,^17^ who advocated for participatory design as a way to improve the usability of apps for chronic condition self-management, the feedback given by the intervention participants provides valuable guidance for further improving the feature's usability. Although 11 of the 12 participants who used ReSound Assist were able to successfully use the feature, they did comment negatively on aspects of the app's effectiveness and efficiency. Specifically, the participants reported that the multiple-choice questions asked by the app—intended to ensure patients fully define the nature, severity, and frequency of their problem—were not always applicable to the problem they were experiencing, thus lessening perceived effectiveness. Several participants reported that as a result, they had to describe their problem in the text box in addition to answering all the questions, thus reducing perceived efficiency. Interestingly, this was most frequently the case when the participant wished to report a problem that could not be addressed through fine-tuning, such as difficulties maintaining a Bluetooth connection or streaming audio input. Together, user feedback and the finding that approximately half of the problems reported via ReSound Assist could not be solved through fine-tuning highlight the possibility of expanding the app's capabilities to increase usability. For example, the app could be programmed to send back automatically generated advice for a range of common problems, such as those related to Bluetooth and audio streaming.

One participant used ReSound Assist to report that her hearing aids were uncomfortable to wear. Such problems almost always require the provider to make physical alterations to the hearing aid, such as changing the ear tip to a different size. This reinforces the important role of face-to-face consultations in hearing health care. However, if an app can help to triage patients such that only those truly requiring face-to-face care attend in-person follow-up appointments, this would still contribute toward alleviating the time and resource burden on individual providers.

There were no significant differences in hearing aid fitting outcomes between the intervention and control groups. This finding suggests that replacing the standard postfitting appointment with an app enabling remote patient-provider communication does not have a detrimental effect on outcomes, at least in the short-term. The current findings are also in agreement with Groth et al.,^26^ who found that remote versus in-person fine-tuning did not have a significant effect on speech understanding in noise or self-reported aided benefit in a sample of 14 adults. However, our results should be considered in the context of several limitations. First, the sample size of this exploratory study was small because its stated goal was to gather preliminary information about a recently introduced app with novel capabilities. It is possible that significant differences between the intervention and control groups may have been detected on one or more of the outcome measures with a larger sample size. On the basis of the present study's findings, a larger trial is warranted. Second, participants were followed for only the first 6 weeks after the hearing aid fitting since outcomes are typically measured at this time point in real-world clinical practice. However, this also means we cannot be certain of the longer-term impact of app-based patient-provider communication. Third, all study participants were experienced hearing aid users with established listening preferences and well-developed hearing aid management skills; those new to hearing aids may present with qualitatively different problems that may or may not lend themselves to resolution via an app. Future work in this area could focus on longer term usage experiences beyond 6 weeks and the ways in which m-health technologies could serve first-time hearing aid users as they acclimatize to amplified sound and acquire the skills necessary to become successful hearing aid users.

## Conclusions

Our study found that (1) ReSound Assist, the remote communication feature in a commercially available hearing aid app, was highly usable based on a validated usability questionnaire and self-report; and (2) replacement of a face-to-face postfitting follow-up appointment with an app did not have a detrimental effect on hearing aid outcomes, at least in the short term. These findings suggest that while there is still scope for improvement, apps enabling patients to communicate remotely with their hearing health care provider are a viable method for experienced hearing aid users to seek and receive help with their hearing aid problems.

## References

[1] Mathers C, Smith A, Concha M Global burden of hearing loss in the year 2000. Geneva: World Health Organization, 2003

[2] World Health Organization. The global burden of disease: 2004 update. Geneva: World Health Organization, 2008

[3] Vos T, Allen C, Arora M, et al. Global, regional, and national incidence, prevalence, and years lived with disability for 310 diseases and injuries, 1990–2015: A systematic analysis for the Global Burden of Disease Study 2015. Lancet 2016**;** 388:1545–1602 27733282 10.1016/S0140-6736(16)31678-6 PMC5055577

[4] Joore MA, van der Stel H, Peters HJM, Boas GM, Anteunis LJC The cost-effectiveness of hearing aid fitting in the Netherlands. Arch Otolaryngol Head Neck Surg 2003**;** 129:297–304 12622538 10.1001/archotol.129.3.297

[5] Chao T-K, Chen TH-H Cost-effectiveness of hearing aids in the hearing-impaired elderly: A probabilistic approach. Otol Neurotol 2008**;** 29:776–783 18725859 10.1097/MAO.0b013e31817e5d1b

[6] Chisolm TH, Johnson CE, Danhauer JL, et al. A systematic review of health-related quality of life and hearing aids: Final report of the American Academy of Audiology task force on the health-related quality of life benefits of amplification in adults. J Amer Acad Audiol 2007**;** 18:151–183 17402301 10.3766/jaaa.18.2.7

[7] Vuorialho A, Karinen P, Sorri M Effect of hearing aids on hearing disability and quality of life in the elderly. Int J Audiol 2006**;** 45:400–405 16938798 10.1080/14992020600625007

[8] Dillon H. Hearing aids, 2nd ed. Sydney: Boomerang Press, 2012

[9] Jenstad LM, Van Tasell DJ, Ewert C Hearing aid troubleshooting based on patients' descriptions. J Amer Acad Audiol 2003**;** 14:347–360 14620609

[10] Angley GP, Schnittker JA, Tharpe AM Remote hearing aid support: The next frontier. J Amer Acad Audiol 2017**;** 28:893–900 29130437 10.3766/jaaa.16093

[11] Timmer BHB, Hickson L, Launer S The use of ecological momentary assessment in hearing research and future clinical applications. Hear Res 2018**;** 369:24–28 29933937 10.1016/j.heares.2018.06.012

[12] Keidser G. Towards ecologically valid protocols for the assessment of hearing and hearing devices. J Amer Acad Audiol 2016**;** 27:502–503 27406657 10.3766/jaaa.27.7.1

[13] Groth J, Bhatt M, Elsig Raun P, Jahn A. Where does the day go? Insights from appointments in a large hearing aid practice. Hear J **2017** Available at https://journals.lww.com/thehearingjournal/blog/onlinefirst/Lists/Posts/Post.aspx?ID=13 (last accessed 430, 2019)

[14] Paglialonga A, Tognola G, Pinciroli F Apps for hearing science and care. Am J Audiol 2015**;** 24:293–298 26649533 10.1044/2015_AJA-14-0093

[15] Clark JL, Swanepoel DW Technology for hearing loss—As we know it, and as we dream it. Disabil Rehabil Assist Technol 2014**;** 9:408–413 24712413 10.3109/17483107.2014.905642

[16] Frøkjær E, Hertzum M, Hornbæk K Measuring usability: Are effectiveness, efficiency, and satisfaction really correlated? *Proceedings of the ACM CHI Conference on Human Factors in Computing Systems*. The Hague, Netherlands: ACM CHI, 2000**;** 345–352

[17] Sarkar U, Gourley GI, Lyles CR, et al. Usability of commercially available mobile applications for diverse patients. J Gen Intern Med 2016**;** 31:1417–1426 27418347 10.1007/s11606-016-3771-6 PMC5130945

[18] Parmanto B, Lewis A, Graham K, Bertolet M Development of the Telehealth Usability Questionnaire (TUQ). Int J Telerehabil 2016**;** 8:3–10 10.5195/ijt.2016.6196 PMC4985278 27563386

[19] Cox RM, Alexander GC The abbreviated profile of hearing aid benefit. Ear Hear 1995**;** 16:176–186 7789669 10.1097/00003446-199504000-00005

[20] Cox RM, Alexander GC Measuring satisfaction with amplification in daily life: The SADL scale. Ear Hear 1999**;** 20:306–320 10466567 10.1097/00003446-199908000-00004

[21] Best V, McLelland M, Dillon H. The BEST (Beautifully Efficient Speech Test) for evaluating speech intelligibility in noise. Presented at the World Congress of Audiology, **2014**, Brisbane, Australia

[22] Keidser G, Dillon H, Mejia J, Nguyen C-V An algorithm that administers adaptive speech-in-noise testing to a specified reliability at any point on the psychometric function. Int J Audiol 2013**;** 52:795–800 23957444 10.3109/14992027.2013.817688

[23] National Health and Medical Research Council. National Statement on Ethical Conduct in Human Research. Canberra, ACT: Commonwealth of Australia, 2007

[24] Myers JL, Well AD, Lorch RF Research design and statistical analysis, 3rd ed. New York: Routledge, 2010

[25] Convery E, Keidser G, Hickson L, Meyer C Factors associated with successful setup of a self-fitting hearing aid and the need for personalized support. Ear Hear 2019**;** 40:794–804 30285978 10.1097/AUD.0000000000000663

[26] Groth J, Dyrlund O, Wagener KC, Meis M, Krueger M Industry research: Fine-tuning outcomes are similar via teleaudiology and face-to-face. Can Audiol 2019**;** 6

